# Phylogeographic Analyses of the East Asian Endemic Genus *Prinsepia* and the Role of the East Asian Monsoon System in Shaping a North-South Divergence Pattern in China

**DOI:** 10.3389/fgene.2019.00128

**Published:** 2019-02-26

**Authors:** Xiangguang Ma, Zhiwei Wang, Bin Tian, Hang Sun

**Affiliations:** ^1^CAS Key Laboratory for Plant Diversity and Biogeography of East Asia, Kunming Institute of Botany, Chinese Academy of Sciences, Kunming, China; ^2^Department of Pharmacy, Guizhou University of Traditional Chinese Medicine, Guiyang, China; ^3^Key Laboratory of Biodiversity Conservation in Southwest China, State Forestry Administration, Southwest Forestry University, Kunming, China

**Keywords:** arid belt, climatic barrier, East Asia, genetic differentiation, genetic diversity, monsoon systems

## Abstract

*Prinsepia* Royle (Rosaceae) is a genus native to China and the Himalayan region. In order to explain its current fragmented distribution pattern and to compare the impact of relatively recent climate changes on the genetic structure of *Prinsepia* species in different regions of China, a total of 66 populations and 617 individuals of four species of *Prinsepia* were genotyped, using three cpDNA markers. Meanwhile, phylogenetic reconstructions and divergence dating were conducted using the cpDNA haplotypes dataset and the nuclear ribosomal internal transcribed spacer (ITS) dataset, respectively. Ecological niche modeling (ENM) was performed to predict the potential distribution of each species of *Prinsepia* at present and during the Last Glacial Maximum. Both ITS and cpDNA gene trees support a north-south divergence of *Prinsepia* species in China. The divergence time of the northern and southern Clades occurred around the late Oligocene epoch. Combining the present distribution of *Prinsepia* species and their habitats, we inferred that the transition to a monsoon climate system in East Asia during the late Oligocene epoch, created a humid forest vegetation zone from central to East China, which potentially gave rise to the north-south divergence of *Prinsepia* species. Both regional climates and allopatric divergence may have played an important role in the speciation of *P. sinensis* and *P. uniflora*. *P. sinensis* had the lowest genetic diversity and a putative northward post-glacial colonization. The distribution range of *P. uniflora* was also extremely sensitive to interglacial-glacial cycles. *P. utilis* from southwestern China preserved more haplotypes than *P. sinensis* and *P. uniflora* due to its multiple and isolated refugia.

## Introduction

**FIGURE 1:**
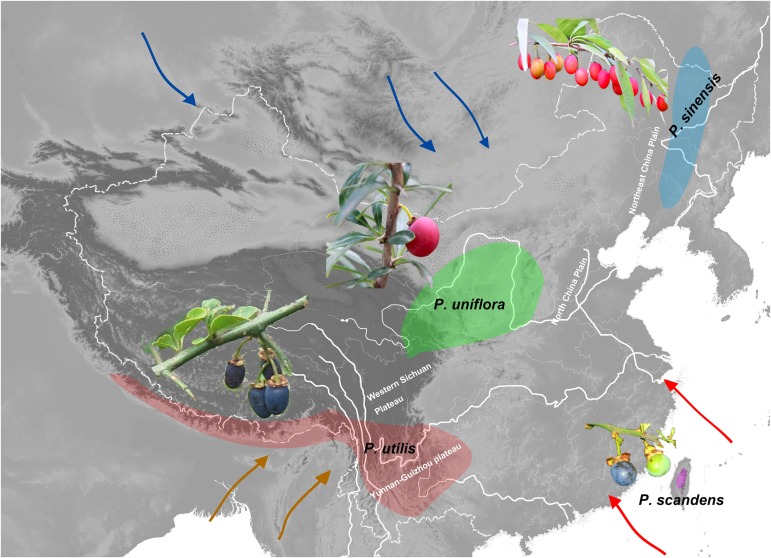
Approximate geographic distributions of the species of *Prinsepia*. Arrows of different colors indicate the East Asian summer monsoon (red), the East Asian winter monsoon (blue), and the Indian summer monsoon (green).

East Asia harbors the most diverse temperate flora in the world, with a vast number (>600) of endemic genera (Wu and Wu, 1996; Manchester et al., 2009). Apart from the uplifts of the Qinghai-Tibet Plateau, the formation of the Asian monsoon system is also thought to have had a profound influence on the evolution and diversification of East Asian flora (Sun and Wang, 2005; Chen et al., 2018). The Asian monsoon system is comprised of the East Asian summer monsoon (EASM), the East Asian winter monsoon (EAWM), and the Indian summer monsoon (ISM) (Figure 1). Although the origin of the three components of the Asian monsoon system was asynchronous, all of them are considered to have existed at least by the late Oligocene epoch (Sun and Wang, 2005; Srivastava et al., 2012). It has been suggested that before the onset of the EASM, there was a broad belt of subtropical arid and semiarid vegetation, extending from east to northwest China (Guo et al., 2008). The eastern and central parts of this arid and semiarid vegetation zone were replaced by a humid forest vegetation zone with the establishment of the East Asian monsoon system, and the arid and semiarid zone became restricted to northwest China (Guo et al., 2008). Multiple changes to aridity or monsoon intensity have occurred in East Asia since then (Sun and Wang, 2005; Wang et al., 2008).

There are a few studies that focused on the origin of different vegetation types or ecosystems in East Asia as a result of East Asian monsoons (e.g., Kong et al., 2017; Yu et al., 2017). However, no studies have focused on the group of species that may have been present in the arid and semiarid vegetation zone in China during the Oligocene epoch. The east–west oriented arid belt is thought to have acted as a climate barrier to forest species between Northeast China and South China (Bai et al., 2016; Ye et al., 2017). Conversely, what is the fate of drought-tolerant or heliophilous plants distributed in this east–west oriented semiarid-arid belt, after the formation of the humid forest vegetation zone which was brought by the East Asian monsoon system?

*Prinsepia* Royle (Rosaceae) is native to China and the Himalayan region. Although the species of this genus exhibit a certain level of habitat divergence, they are all drought-tolerant shrubs, which have axillary spines and bear blossoms in early spring. This genus contains four species which are found in four separate areas (Figure 1; Gu and Bartholomew, 2003). Among them, *P. uniflora* Batalin is found in the semiarid region of north and northwest China, mainly on the Loess plateau (Figure 1). *P. sinensis* (Oliv.) Oliv. ex Bean is morphologically similar to *P. uniflora*, but this species is found in the eastern part of northeast China, an area more affected by the EASM (Figure 1). *P. utilis* Royle is found from southwest China to the Himalayan region (Figure 1) and the western Yunnan-Guizhou plateau is the core distribution area of this species. *P. scandens* Hayata, which only appears in the mountain ranges of mid-eastern Taiwan (Figure 1), is morphologically very similar to *P. utilis*.

Because different plant genera differ in their evolutionary ages and niche evolution, their divergence or genetic structure should be affected by different geological processes and climatic events. From previous large-scale phylogenetic studies of Rosaceae, we supposed that the time of origin for *Prinsepia* pre-dated the onset of the East Asian monsoon system (Xiang et al., 2017; Zhang et al., 2017). In this study, we first wanted to elucidate the phylogenetic relationships among *Prinsepia* species, which exhibit a very particular fragmented distribution. The gap between the two northerly species and the two southerly species in this genus is exactly the core region of the temperate and subtropical forests in East Asia (Figure 1). We aimed to identify geological or climatic events behind the fragmented distribution within this genus. In addition, although quite a lot of phylogeographic studies have focused on plants in north and south China and their responses to the glacial-interglacial cycles of the Quaternary, phylogeographic studies of endemic genera of East Asia, containing several species, are rare. This research involved a phylogeographic study of all the species of *Prinsepia* and we aimed to compare the impact of the relatively recent environmental changes (Quaternary glaciations) on the genetic structure of each species in the different regions of China.

## Materials and Methods

### Plant Material

We sampled all the four species of *Prinsepia*, covering most of its distribution range in China. A total of 617 individuals from 66 populations were sampled, including 18 populations of *P. uniflora*, six populations of *P. sinensis*, 39 populations of *P. utilis*, and three populations of *P. scandens*. Two to fifteen individuals were collected from each population. The total numbers of individuals sampled of *P. uniflora*, *P. sinensis*, *P. utilis*, and *P. scandens* were 145, 61, 399, and 12, respectively. Silica-gel dried plant materials were all collected from the wild. The collection locations for each population are presented in Supplementary Appendix 1.

### DNA Isolation, Amplification, and Sequencing

Total genomic DNA was extracted using a plant genomic DNA kit according to the manufacturer’s instructions (Tiangen, Beijing, China). We sequenced three intergenic spacer (IGS) regions of cpDNA (*psbA-trnH*, *psbK-psbI*, *psbJ-petA*) for phylogeographic analyses. Because the nuclear ribosomal internal transcribed spacer (ITS) region lacked variations within each species and between *P. utilis* and *P. scandens*, we sequenced five, four, six, and three samples for *P. uniflora*, *P. sinensis*, *P. utilis*, and *P. scandens*, respectively. The primer sequences and procedures for DNA amplification are listed in Table 1. All PCR products were sent to Sangon Biotech (Shanghai, China) for sequencing, and were sequenced using amplification primers. All sequences were submitted to GenBank (accession numbers: MK442012–MK442070).

**Table 1:** The primer sequences and the corresponding references.

Locus	PCR primers (5′–3′)	Reference
ITS	ITS4: TCCTCCGCTTATTGATATGC	White et al., 1990
	ITS5: GGAAGTAAAAGTCGTAACAAGG	
*PsbA-trnH*	*PsbA*:GTTATGCATGAACGTAATGCTC	Sang et al., 1997
	*trnH*:CGCGCATGGTGGATTCACAAATC	
*PsbK-psbI*	*psbK*: TTAGCCTTTGTTTGGCAAG	Lahaye et al., 2008
	*psbI*: AGAGTTTGAGAGTAAGCAT	
*psbJ-petA*	*psbJ*: ATAGGTACTGTARCYGGTATT	Shaw et al., 2007
	*petA*: AACARTTYGARAAGGTTCAATT	

### Phylogeographic Analyses of cpDNA

We combined three cpDNA fragments for the phylogeographic analyses. Fourteen indels (Gaps) were treated as a single mutation event and coded as substitutions A or T (a third base G or C will be used when three kinds of mutations coexisted). However, we deleted a 24 bp and a 65 bp region (aligned length) from the *psbA-trnH* data matrix, because they contained complex indel information and were too divergent to be aligned.

The number of cpDNA haplotypes (h), haplotype diversities (Hd) and nucleotide diversities (p) for each species were calculated using DNAsp V5.1 (Librado and Rozas, 2009). Based on the phylogenetic relationship, NETWORK v.5 (Fluxus Technology Ltd.) was used to generate the median-joining network for *P. uniflora* and *P. sinensis*, *P. utilis* and *P. scandens*, respectively. Permut 1.0^1^ was used to compare Gst and Nst of *P. uniflora* and *P. utilis*, with 1000 permutations.

### Divergence Dating

Bayesian phylogenetic reconstructions and divergence time estimations were conducted independently for concatenated cpDNA haplotypes and the ITS dataset, using Beast v1.8.4 (Drummond and Rambaut, 2007). We selected *Exochorda serratifolia* and *Oemleria cerasiformis* as outgroups for the cpDNA tree divergence time estimation, as used in previous large-scale phylogenetic studies of Rosaceae (Xiang et al., 2017; Zhang et al., 2017; Supplementary Appendix 2). The sequences for Sorbarieae and Kerrieae were also included in the ITS tree analysis (Supplementary Appendix 2). We used the original cpDNA sequences of all four species (without recoding and deleting two complex regions from *psbA-trnH*) to generate a haplotype file and these haplotypes were used for the divergence time estimation of cpDNA. The fossil records for *Oemleria janhartfordae* and *Neviusia* sp. from North America were used for calibration (DeVore et al., 2004; Benedict et al., 2011; Xiang et al., 2017). We used fossil constraint age, as provided by Xiang et al. (2017), and a standard deviation of 1.0 to calibrate the corresponding nodes. The MrModeltest 2.3 (Nylander, 2004) was used to determine the best-fitting nucleotide substitution model for the two datasets. The best-fit models for the ITS and cpDNA dataset were all GTR + G. An uncorrelated lognormal, relaxed clock model was applied to estimate rate change (Drummond et al., 2006). The birth-death process and Yule process were run independently as the tree priors. Marginal likelihood estimation, using path sampling and stepping-stone sampling, was used to perform model selection (Baele et al., 2012). Convergence of all parameters was checked using Tracer v1.7 (Rambaut et al., 2018). The analyses were run for 100 million generations and sampled every 1000 generations.

### Ecological Niche Modeling

In order to study the distribution range shifts of each species from the Last Glacial Maximum (LGM; c. 21 kya) to the present, we carried out ecological niche modeling (ENM) using Maxent 3.2 (Phillips et al., 2006) with the default parameters. *P. utilis* and *P. scandens* were treated as a complex in this analysis because of their close phylogenetic relationship and similar habitat. The distribution data for each species were obtained from our collection and the herbarium records ^2^^,^^3^ . After removing duplicates, we identified 18 sites for *P. sinensis*, 35 sites for *P. uniflora* and 68 sites for *P. utilis* and *P. scandens* (Supplementary Figure S1). The current and LGM climate data for 19 BIOCLIM variables were downloaded from the WorldClim database ^4^. We chose the highest resolution [30 arc-s (∼1 km)] for the current data and the Community Climate System Model 3.0 at 2.5 arc-min resolution for the LGM. Strongly correlated climatic variables, according to Pearson’s correlation coefficient (*r* > 0.9), were excluded (Supplementary Appendix 3). All models were run with 20 replicates in Maxent. Around 25% of the data in each run was randomly chosen for model testing and 75% was chosen for model training.

### Climatic Data Analysis

In order to compare the difference in climate, between the distribution areas of adjacent species pairs (*P. sinensis* and *P. uniflora*; *P. uniflora* and *P. utilis*), we extracted the 19 BIOCLIM variables under current conditions (∼1960–1990) at the highest resolution [30 arc-s (∼1 km)] (Hijmans et al., 2005) using ArcGIS 10.2^5^. The extracted locations were the same as the collection records in the ENM for *P. sinensis* and *P. uniflora*. However, we only used the distribution data for *P. utilis* on the Yunnan-Guizhou Plateau. We used a two-tailed *t*-test in Excel to compare the difference for each of the BIOCLIM variables at the sites of each pair of species.

## Results

### Bayesian Phylogenetic Analyses and Molecular Dating

**FIGURE 2:**
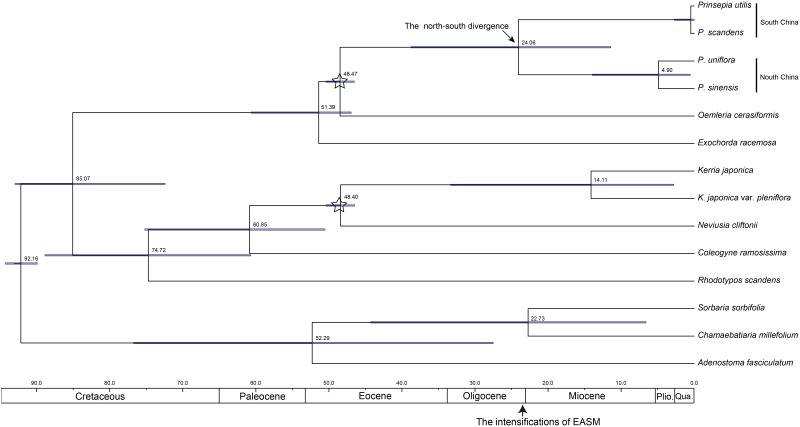
The chronogram for *Prinsepia* derived from BEAST analysis of ITS sequences. Positions of the fossil calibrations are indicated by a five-pointed star. Divergence times are labeled on each node. Blue bars at nodes represent the 95% highest probability density (HPD) for the age of that node.

**FIGURE 3:**
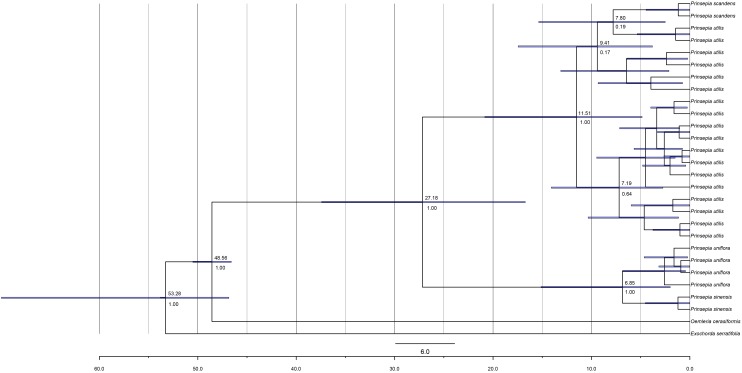
The chronogram of *Prinsepia* derived from BEAST analysis of combined cpDNA sequences.

Both the ITS tree and the cpDNA tree supported a north-south divergence of *Prinsepia* species. *P. uniflora* and *P. sinensis* formed one clade, while *P. utilis* formed another monophyletic clade with *P. scandens* (Figure 2, 3). The populations of *P. uniflora* and *P. sinensis* each formed a monophyletic clade. In contrast, *P. scandens* did not form a monophyletic clade but was embedded in the clade of *P. utilis* (Figure 2, 3 and Supplementary Figure S2).

The birth-death (BD) process was chosen as the best tree prior, based on the marginal likelihood estimation (MLE), using path sampling and stepping-stone sampling (MLEBD = −3041.00 and −3041.06, MLEYULE = −3041.86 and −3041.87 in ITS analysis; MLEBD = −3038.82 and −3038.82, MLEYULE = −3042.35 and −3042.40 in cpDNA analysis). The median origin time for *Prinsepia* was around 48.60 Mya, based on *Oemleria*, which is the existing genus most closely related to *Prinsepia*. The mean divergence time for the north and south clades of *Prinsepia* was 24.06 Mya (95% HPD = 11.41–38.75) in the ITS tree (Figure 2) and 27.18 Mya (95% HPD = 16.72–37.42) in the cpDNA tree (Figure 3). Within the North China clade, the mean divergence time for *P. uniflora* and *P. sinensis* was 4.90 Mya in the ITS tree and 6.85 Mya in the cpDNA tree (Figure 2, 3). Because of the lack of variation between *P. utilis* and *P. scandens*, in terms of their ITS sequences, we were unable to determine a divergence time for these two species. In the cpDNA tree, *P. scandens* did not, in fact, form a separate branch but was embedded within the clade of *P. utilis*.

### Genetic Diversity and Genetic Structure

After recoding the indels and deleting two regions from the *psbA-trnH* matrix, we obtained a concatenated cpDNA dataset with a length of 1432 bp. The basic genetic data for the four *Prinsepia* species is shown in Table 2.

**Table 2:** Genetic diversity assessment for four *Prinsepia* species using the concatenated cpDNA data set.

Species	No. of samples	No. of haplotypes	Hd	π
*Prinsepia sinensis*	61	2	0.064	0.00009
*Prinsepia utilis*	399	18	0.843	0.004
*Prinsepia uniflora*	145	6	0.775	0.00118
*Prinsepia scandens*	12	2	0.303	0.00021

**FIGURE 4:**
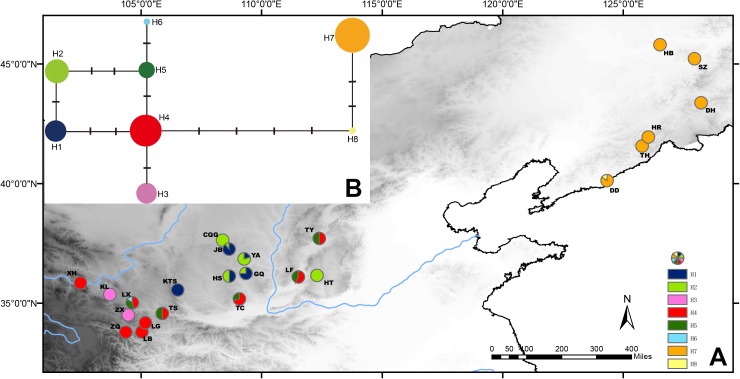
**(A)** Sampling localities and distribution of cpDNA haplotypes of *P. uniflora* and *P. sinensis*. **(B)** Median-joining network of the cpDNA haplotypes of *P. uniflora* and *P. sinensis*.

The population genetic structure of *Prinsepia* in north China is simple (Figure 4). Only two haplotypes were identified from 61 individuals of *P. sinensis*. All the populations of *P. sinensis* share an identical haplotype and only the Dandong (DD) population has an additional haplotype (Figure 4). Although six cpDNA haplotypes were identified from *P. uniflora*, these haplotypes were closely related (Figure 4). No significant phylogeographic structure was found for *P. uniflora* (Nst > Gst, *p* > 0.05).

**FIGURE 5:**
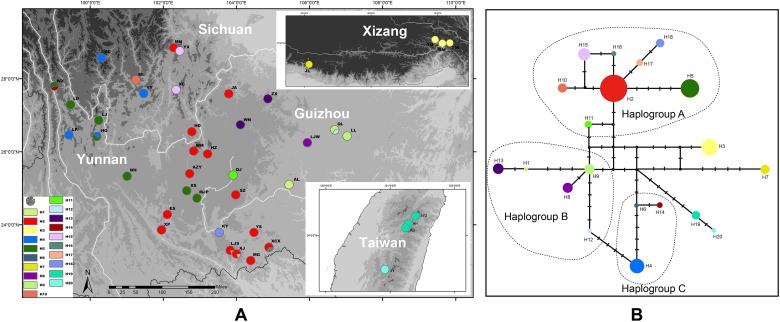
**(A)** Sampling localities and distribution of cpDNA haplotypes of *P. utilis* and *P. scandens*. **(B)** Median-joining network of the cpDNA haplotypes of *P. utilis* and *P. scandens*.

Twelve individuals of *P. scandens* were found to have two haplotypes (Figure 5), and these two haplotypes are closely related, and have seven nucleotide differences compared to the closest existing haplotype of *P. utilis*, according to the network analysis (Figure 5). The most closely related haplotype was found in the easternmost part of the distribution region of *P. utilis* in mainland China. A maximum of 18 haplotypes were identified from 399 individuals of *P. utilis* (Figure 5). *P. utilis* exhibited the highest haplotype diversity (0.843) and nucleotide diversity (0.004). Like the haplotypes from Taiwan, two haplotypes from Xizang and the other haplotypes of *P. utilis* were also connected by several hypothetical missing haplotypes (Figure 5). Permut did not reveal a significant phylogeographic structure for *P. utilis* (Nst > Gst, *P* > 0.05). However, a north-south oriented distribution pattern was found for several haplotypes in areas of the Hengduan mountains and Yunnan-Guizhou Plateau (Figure 5).

### Ecological Niche Modeling

**FIGURE 6:**
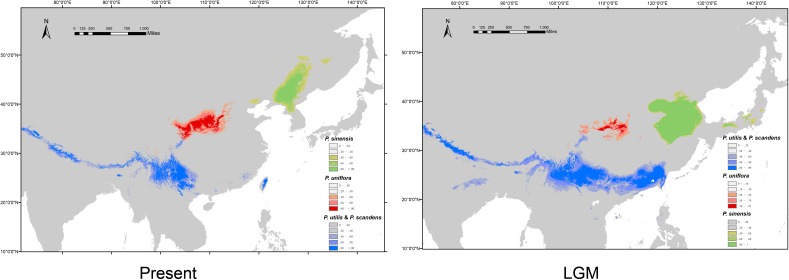
Potential distribution probability of occurrence of the species of *Prinsepia* at present (1970–2000) and during the Last Glacial Maximum (LGM: c. 21 kya).

We obtained high AUC (the Area Under the Curve) values (>0.97) for each species and climate scenario, suggesting model performance of ENM are reliable. The predicted distribution range for *P. sinensis* during the LGM was more southerly than it is at present (Figure 6). *P. uniflora* had a more restricted distribution during the LGM than it does at present, and the range was also slightly south of the current distribution (Figure 6). The predicted species distribution during the LGM for *P. utilis* and *P. scandens* was significantly larger than the predicted species distributions under current conditions (Figure 6).

### Climatic Data Analysis

The means of all but four (bio2, bio5, bio8, and bio10) of the BIOCLIM variables were significantly different (*P* < 0.01) when the sites of *P. uniflora* and *P. sinensis* were compared (Table 3). There were no statistically significant differences in mean values (*P* > 0.05) for three BIOCLIM variables (bio8, bio10, bio15) when comparing the ranges of *P. uniflora* and *P. utilis* (Table 3).

**Table 3:** Results of *t*-tests on the 19 BioClim factors of adjacent species pairs.

	bio1	bio2	bio3	bio4	bio5	bio6	bio7	bio8	bio9	bio10	bio11	bio12	bio13	bio14	bio15	bio16	bio17	bio18	bio19
*P. utilis* vs. *P. uniflora*	0.00	0.00	0.00	0.00	0.00	0.00	0.00	**0.68**	0.00	**0.08**	0.00	0.00	0.00	0.00	**0.90**	0.00	0.00	0.00	0.00
*P. uniflora* vs. *P. sinensis*	0.00	**0.06**	0.00	0.00	**0.28**	0.00	0.00	**0.20**	0.00	**0.45**	0.00	0.00	0.00	0.00	0.00	0.00	0.00	0.00	0.00

*Significantly different: P < 0.05; P-values >0.05 were given in bold print. The Description of the bioclimatic variables can be found at the website (http://worldclim.org/bioclim).*

## Discussion

### The North-South Divergence of *Prinsepia* Shaped by the EASM and the Fragmented Distribution of *Prinsepia* in North and South China

**FIGURE 7:**
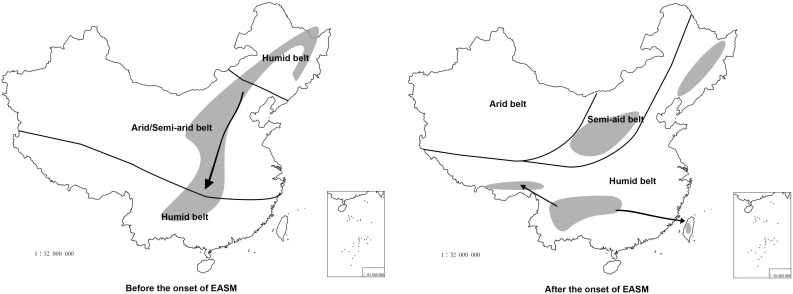
The putative distribution of *Prinsepia* before and after the onset of the EASM. The climate zones follow Guo et al. (2008). Thick black lines represent boundaries of different climatic zones and arrows represent dispersal routes.

*Oemleria* is a monotypic genus endemic to western North America; this is the existing genus most closely related to *Prinsepia* (Xiang et al., 2017). Because the reliable fossil of *Oemleria* has been dated to late in the early Eocene epoch (49.42 ± 0.54 Ma) (Benedict et al., 2011), the origin time for *Prinsepia* should not follow after this time, if no other group had become extinct. All the related genera of *Prinsepia*, including *Oemleria*, *Exochorda* Lindl., *Neviusia* A. Gray, *Kerria* Candolle, *Rhodotypos* Siebold and Zuccarini, and *Coleogyne* Torrey, are all temperate taxa, which are distributed in East Asia or North America. These existing temperate taxa or their ancestors in East Asia or North America had a more northerly distribution in the Paleogene epoch, i.e., Arcto-Tertiary Flora (Milne and Abbott, 2002), so *Prinsepia* probably diverged from *Oemleria* in a high or middle-high latitude area and spread southward gradually along the mountain chains of China (Figure 7).

All four *Prinsepia* species have axillary spines, indicating that the ancestral species of *Prinsepia* may have grown in semi-arid areas. *P. uniflora* is a drought-tolerant shrub that grows in the semiarid zone of northwest China. Although *P. utilis* exhibited a certain degree of habitat divergence from *P. uniflora* (Table 3), it still cannot grow in humid and dense forests. *P. utilis* tends to grow in full sun and well-drained areas and blooms in the driest season (early spring). Descendent species are likely to occupy more or less the same ecological niches as their ancestor. From the current habitats of *P. uniflora* and *P. utilis*, which are found on opposite sides of central China, we infer that the ancestral species of *Prinsepia* was likely to have occurred (or survived) in the semiarid vegetation zone of central China during the Oligocene epoch (Figure 7).

The divergence time (median) of the northern clade and the southern clade of *Prinsepia* was the late Oligocene epoch in both cpDNA and ITS trees, which is very close to the onset or intensification of the EASM (Sun and Wang, 2005). With the influence of the East Asian monsoon circulation system from the late Oligocene epoch onward, eastern, and central China transformed into a humid forest vegetation zone (Guo et al., 2008; Yu et al., 2017). From the current distribution of *P. utilis* and *P. uniflora* we can see that although the mountainous environment of the eastern intervening area between these two species can provide suitable altitudes for *Prinsepia* plants, this region is exactly the core region of the temperate and subtropical forests of East Asia, which does not contain a large area of suitable habitat for *Prinsepia* species. The western intervening area, where *Prinsepia* does not occur now, is the eastern part of the Qinghai-Tibet Plateau, i.e., the Western Sichuan Plateau (Figure 1). Although the result of the ENM showed that the eastern margin of the Western Sichuan Plateau has conditions suitable for *P. utilis* under both current and LGM climate models, *P. utilis* actually only has a northern presence in southwest Sichuan (Figure 1, 7). Therefore, the ancestral species of *Prinsepia*, which gave rise to the north-south divergence of this genus, was more likely found in the mountain chains of central China rather than western Sichuan (Figure 7).

After the establishment of the EASM during the Oligocene epoch, the dry belt within China migrated northward (Guo et al., 2008; Figure 7). *P. uniflora* is mainly found in the semiarid zone of north and northwest China and this species is also the most drought-tolerant of all the members of the genus (Gu and Bartholomew, 2003). Although the Western Yunnan-Guizhou Plateau is located at the meeting point of the Indian and East Asia summer monsoons (Figure 1) and the forest vegetation in this area is well-developed, this region is famous for its winter and spring drought, as well as plentiful sunlight. More importantly, this region has a great variety of landscapes, providing various habitats for different plants. Many drought-tolerant or heliophilous shrubs can be found here and many drought-tolerant temperate genera have been found to have evolved distinct species in this region, for example *Cotinus nana* (Min and Barfod, 2008), *Ostryopsis nobilis* (Li and Skvortsov, 1999), and *Pistacia weinmanniifolia* (Min and Barfod, 2008).

If niche conservatism contributed greatly to the initial north-south divergence of *Prinsepia*, local adaptation may have had an influence on the subsequent speciation of *P. sinensis* and *P. uniflora*. From the climate data analysis, we found that all the bioclimatic variables related to precipitation, showed significant differences between the sites where *P. sinensis* and *P. uniflora* were found. In contrast, four bioclimatic variables relating to temperature, especially the temperature of growing season (the wettest Quarter and warmest Quarter), showed no significant difference between these two species (Table 3). *P. sinensis* is found in the eastern part of northeast China, which is more influenced by the EASM. The divergence time of *P. sinensis* and *P. uniflora* was from the late Miocene to the Pliocene. It has been suggested that the EASM intensified in the late Miocene–Pliocene (Ao et al., 2016), which may relate to the divergence of *P. sinensis* and *P. uniflora*. An alternative scenario is that ecological divergence was behind the allopatric speciation of these two species. Allospecies always differ in their ecological niches to some extent, reflecting adaptations to different climatic regimes or different competitive abilities (Rundell and Price, 2009). The Northeast China Plain and North China Plain may have played an important role in the obstruction of gene flow between these two species during different geological periods (Figure 1). And from the results of ENM, although the predicted distribution of *P. sinensis* was much more southerly in the LGM than its current distribution, it is still confined to the eastern area and has experienced increasing isolation from *P. uniflora*.

At least based on the results of molecular analyses, *P. scandens* may not be an independent species, but may be just an evolutionary lineage of *P. utilis*. The morphological characters of *P. scandens* are also very similar to *P. utilis*. A total of 50 genera of seed plants exhibit a disjunct distribution between the island of Taiwan and southwest mainland China (Ye et al., 2012). The divergence time between *P. scandens* and the populations of *P. utilis* could not be determined from the cpDNA estimation because the corresponding node was only weakly supported. Meanwhile, no variation was found between the ITS sequences of *P. scandens* and *P. utilis* from southwest China. Although it is difficult to explain why such a huge discrepancy existed between the mutation rate of the ITS region and cpDNA fragments, we are inclined to believe that the divergence time for *P. scandens* and *P. utilis* is relatively recent. *P. scandens* may originate from a dispersal event from southwest China. From the ENM analysis, we find that the suitable ranges during the LGM for *P. utilis* and *P. scandens* were larger than under current conditions and these two species may have had contact with each other during the LGM. This result is similar to some cold tolerant plants which have disjunct distributions between southwest China and Taiwan (Gao et al., 2015; Niu et al., 2017). *P. scandens* is found in areas of high elevation in Taiwan. The climate of south China became cold and dry during the LGM, thus providing a large area of suitable habitats for *P. utilis* and *P. scandens*.

### Genetic Diversity and Geographic Structure of *Prinsepia* Species in North and South China

*Prinsepia sinensis* has only two haplotypes based on three cpDNA fragments in our study. One of these was shared by all six populations and the other haplotype was only found in the DD population, which is located in the southernmost part of the species’ range (Figure 4). Although phylogeographical analyses of some species have supported the suggestion that potential refugia existed in northeast China during glacial periods (Qiu et al., 2011; Zhao et al., 2013), *P. sinensis* probably retreated south area at this time, as suggested by its unique haplotype in northern areas and the ENM result; this situation is similar to the post-glacial recolonization of European biota (Hewitt, 1999).

A relatively high level of genetic diversity was observed in *P. uniflora*, which may be the result of the broad west to east range of this species. Perhaps because no large geographical barriers exist in this region, the haplotypes of *P. uniflora* have relatively close relationships with each other and no phylogeographical structure was found. *P. uniflora* mainly grows on the Loess plateau and prefers a semiarid habitat and its distribution during the LGM was much smaller than its current range, according to the ENM result (Figure 6). This suggests that although the drought resistant plants on the Loess plateau were able to retreat to southern areas during the LGM, they may still not have been able to cross climatic barriers such as the Ta-pa Mountains, where precipitation is plentiful and there are well-developed forests because of the EASM.

Southwest China is not only well-known for its high species richness, but is also characterized by the high degree of genetic diversity that it hosts (Sun et al., 2017). This high diversity can be attributed to a combination of long-term isolation between different mountain systems and environmentally stable habitats during the Pleistocene epoch (Qu et al., 2014). *P. utilis* has a higher haplotype diversity than the two northern species and a north-south oriented distribution pattern was found for several haplotypes (H2 and H5) (Figure 5), which may be closely related to the north-to-south orientation of the mountains in southwest China, and historical river separation (Zhang et al., 2011). The north-south features of southwest China created barriers to gene flow, as demonstrated for many groups (Liu et al., 2013; Feng et al., 2016; Luo et al., 2017).

*Prinsepia utilis* not only has the highest genetic diversity, but was also found to exhibit large genetic differentiation across the whole distribution range. The two haplotypes from Xizang were connected with the haplotypes from YGP by several hypothetical missing haplotypes (Figure 5B). *P. utilis* is distributed in a long and narrow area along the Himalayas. Many species with such a distribution have been found to exhibit allopatric divergence from conspecific populations in the Hengduan Mountains region (Jia et al., 2011; Li et al., 2011), because gene flows between them can be easily blocked by north-south oriented mountains or allopatric fragmentation caused by climate oscillations. Although no significant phylogeographic structure was found for *P. utilis* in our Permut analysis, the haplotypes of *P. utilis* in the Hengduan Mountains and YGP can be divided into three haplogroups according to their phylogenetic relationship and geographical distribution (Figure 5B). Several haplotypes (H2, H5, H10–11, H15–18) from southwest Sichuan, western Yunnan and central areas of Yunnan constituted a haplogroup (haplogroup A). An ancestral haplotype (H2) can be identified in this haplogroup, and this haplotype also has the widest distribution. The other haplotypes were all derived from this haplotype and showed fewer variations from it. In addition, the derived haplotypes were all found in regions adjacent to the range of haplotype H2. Perhaps because this region is the most suitable habitat for *P. utilis* according the ENM result, the ancestral haplotype experienced historical range expansion and subsequent isolation, which created the new haplotypes. The haplotypes from eastern Yunnan and Guizhou (H1, H8–9, and H12–13) formed haplogroup B. Haplogroup C contained three haplotypes form western Yunnan (H4, H6, and H14). Haplogroup B was found to have a close relationship to both haplogroups A and C, and haplogroup C showed a closer relationship to haplogroup B. Haplogroups B and C are geographically isolated, and the intervening areas are occupied by haplotypes from haplogroup A. Given these facts, we believe that the current phylogeographic structure of *P. utilis* can be attributed to the allopatric divergence of the ancestral lineage (i.e., haplogroups B, C, the haplotypes from Xizang and *P. scandens*), and regional range expansion of the renascent lineage (haplogroup A). Meanwhile, secondary contacts between the derived lineage and the remnant lineage were also found in two populations of *P. utilis* in northwest Yunnan (HQ and WX) (Figure 5A).

## Conclusion

This study revealed that the humid belt in central-eastern China which was created by the EASM in the late Oligocene epoch might have acted as a climatic barrier for drought-tolerant plants. After a north-south divergence of *Prinsepia*, drastic genetic divergence occurred within the northern or southern clade. From our phylogeographic results, we infer that the distribution ranges of the plants in north China seem to have been highly impacted by the glacial interglacial cycles, resulting in this region hosting lower genetic diversity than southwest China. In contrast, southwest China retained more haplotypes due to its multiple and independent refugia, with the genetic divergence closely related to the high mountain and deep valley systems and the environmental conditions in southwest China.

## Author Contributions

HS and BT conceived the study. BT, ZW, and XM collected the samples. BT completed the experiments. XM and BT analyzed the data. XM was the lead writer. BT, ZW, and HS corrected the manuscript.

## Conflict of Interest Statement

The authors declare that the research was conducted in the absence of any commercial or financial relationships that could be construed as a potential conflict of interest.

## Footnotes

1 http://www.pierroton.inra.fr/genetics/labo/Software/Permut/

2 www.cvh.ac.cn

3 www.gbif.org

4 www.worldclim.org

5 https://www.arcgis.com
