# Supplementary material for: Phylogeographic Analyses of the East Asian Endemic Genus Prinsepia and the Role of the East Asian Monsoon System in Shaping a North-South Divergence Pattern in China
Source: Front Genet. 2019 Feb 26;10:128. doi: 10.3389/fgene.2019.00128 (PMC6399409; doi:10.3389/fgene.2019.00128)
Supplement: Supplementary file 1 [file Data_Sheet_1.docx]

Supplementary Material

Phylogeographic analyses of the East Asian endemic genus *Prinsepia* and the role of the East Asian monsoon system in shaping a north-south divergence pattern in China

**Xiangguang Ma^1^, Zhiwei Wang^2^,** **Bin Tian^1,3*^,** **Hang Sun^1*^**

* Correspondence:

Bin Tian

[tianbinlzu@163.com](mailto:tianbinlzu@163.com)

Hang Sun

[sunhang@mail.kib.ac.cn](mailto:sunhang@mail.kib.ac.cn)

# Supplementary Figures and Tables

## Supplementary Figures


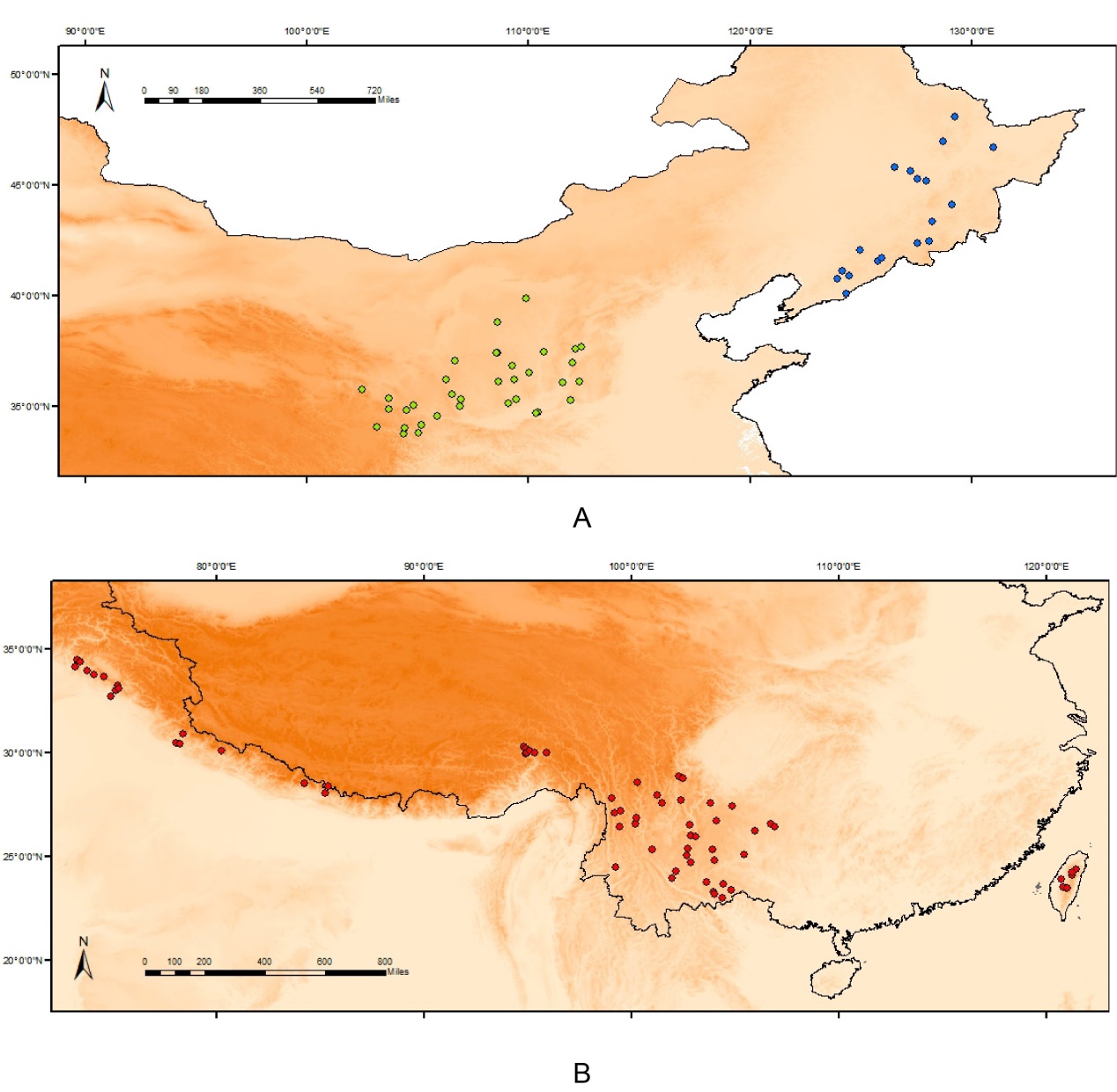


Figure S1 | The species occurrence points of *Prinsepia* species for species distribution modeling; blue: *P. sinensis*, green: *P. uniflora*, red: *P.* *utilis* and *P. scanden*s.


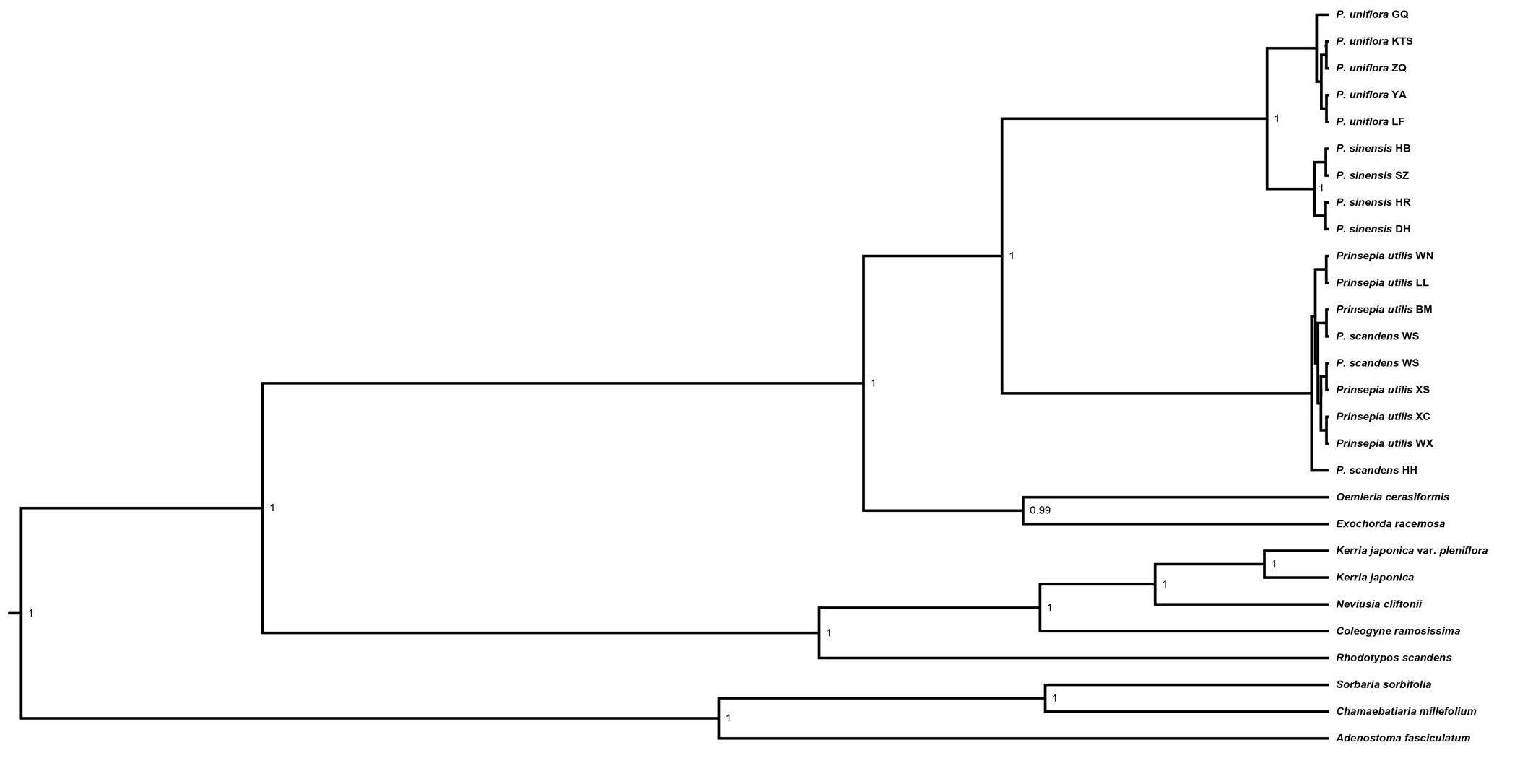


Figure S2 | Bayesian tree derived from BEAST analysis of the ITS dataset (Bayesian posterior probabilities < 0.5 are not shown at nodes).

## Supplementary Tables

Appendix 1 Sample locations, sample sizes, genetic diversities and chlorotypes in each populations of *Prinsepia*.

| species | code | Sample size | location | Lon. | Lat. | Haplotypes |
| --- | --- | --- | --- | --- | --- | --- |
| **North China** |  |  |  |  |  |  |
| *P. sinensis* | DD | 13 | Dandong, Liaoning | 124.33 | 40.12 | H7, H8 |
| *P. sinensis* | DH | 14 | Dunhua, Jilin | 128.23 | 43.37 | H7 |
| *P. sinensis* | HB | 10 | Harbin, Heilongjiang | 126.53 | 45.80 | H7 |
| *P. sinensis* | HR | 7 | Huanren, Liaoning | 125.78 | 41.57 | H7 |
| *P. sinensis* | SZ | 8 | Shangzhi, Heilongjiang | 127.96 | 45.21 | H7 |
| *P. sinensis* | TH | 9 | Tonghua, Jilin | 125.93 | 41.73 | H7 |
| *P. uniflora* | CQG | 10 | Jingbian, Shaanxi | 108.60 | 37.45 | H2 |
| *P. uniflora* | GQ | 8 | Ganquan, Shaanxi | 109.35 | 36.25 | H1,H2 |
| *P. uniflora* | HT | 11 | Hongtong, Shanxi | 112.30 | 36.16 | H2 |
| *P. uniflora* | HS | 6 | Heshui, Gansu | 108.66 | 36.13 | H1, H2 |
| *P. uniflora* | JB | 9 | Jingbian, Shaanxi | 108.57 | 37.46 | H1, H2 |
| *P. uniflora* | KL | 11 | Kangle, Gansu | 103.70 | 35.37 | H3 |
| *P. uniflora* | KTS | 5 | Kongtongshan, Gansu | 106.52 | 35.56 | H1 |
| *P. uniflora* | LF | 7 | Linfen, Shanxi | 111.52 | 36.10 | H4, H5 |
| *P. uniflora* | LB | 6 | Leiba, Lixian, Gansu | 105.03 | 33.80 | H4 |
| *P. uniflora* | LG | 5 | Liugou, Lixian, Gansu | 105.18 | 34.19 | H4 |
| *P. uniflora* | LX | 14 | Longxi, Gansu | 104.63 | 35.05 | H4, H5, H6 |
| *P. uniflora* | TC | 7 | Tongchuan, Shaanxi | 109.08 | 35.18 | H4, H5 |
| *P. uniflora* | TS | 8 | Tianshui, Gansu | 105.89 | 34.57 | H4, H5 |
| *P. uniflora* | YA | 5 | Yanan, Shaanxi | 109.28 | 36.85 | H1,H2 |
| *P. uniflora* | TY | 4 | Tianlongshan, Taiyuan, Shanxi | 112.39 | 37.71 | H4, H5 |
| *P. uniflora* | XH | 11 | Xunhua, Qinghai | 102.48 | 35.85 | H4 |
| *P. uniflora* | ZQ | 9 | Zhouqu, Gansu | 104.37 | 33.79 | H4 |
| *P. uniflora* | ZX | 12 | Zhangxian, Gansu | 104.47 | 34.51 | H3 |
| **South China** |  |  |  |  |  |  |
| *P. scandens* | HH | 3 | Hehuanshan, Taiwan | 121.28 | 24.18 | H20 |
| *P. scandens* | WS | 4 | Renai, Nantou, Taiwan | 121.24 | 24.12 | H20 |
| *P. scandens* | XY | 2 | Xinyi, Nantou, Taiwan | 120.92 | 23.47 | H21 |
| *P. scandens* | SYZ | 3 | Yilan, Taiwan | 121.40 | 24.30 | H20 |
| *P. utilis* | AL | 2 | Anlong, Guizhou | 105.44 | 25.10 | H1 |
| *P. utilis* | AZY | 9 | Aziying, Kunming, Yunnan | 102.72 | 25.40 | H2 |
| *P. utilis* | BM | 15 | Bomi, Xizang | 95.34 | 29.99 | H3 |
| *P. utilis* | DC | 12 | Daocheng, Sichuan | 100.30 | 28.58 | H4 |
| *P. utilis* | ES | 6 | Eshan, Yunnan | 102.11 | 24.28 | H2 |
| *P. utilis* | HD | 14 | Huidong, Yunnan | 102.78 | 26.55 | H2 |
| *P. utilis* | HQ | 14 | Heqing, Yunnan | 100.18 | 26.56 | H4, H5, H6 |
| *P. utilis* | HZ | 10 | Huize, Yunnan | 103.10 | 25.94 | H2 |
| *P. utilis* | JA | 3 | Jingan, Zhaotong, Yunnan | 103.79 | 27.59 | H2 |
| *P. utilis* | JL | 12 | Jilong, Xizang | 85.36 | 28.39 | H7 |
| *P. utilis* | MJP | 13 | Majinpu, Chengjiang, Yunnan | 102.86 | 24.73 | H5 |
| *P. utilis* | LD | 16 | Ludian, Lijiang, Yunnan | 99.46 | 27.18 | H5 |
| *P. utilis* | LJ | 5 | Lijiang, Yunnan | 100.23 | 26.86 | H5 |
| *P. utilis* | KY | 10 | Kaiyuan, Yunnan | 103.62 | 23.79 | H18 |
| *P. utilis* | LJS | 15 | Laojunshan, Wenshan, Yunnan | 103.93 | 23.30 | H2, H17 |
| *P. utilis* | LJW | 15 | Longjiawan, Anshan, Guizhou | 105.94 | 26.25 | H8 |
| *P. utilis* | LL | 11 | Longli, Guizhou | 106.90 | 26.43 | H9 |
| *P. utilis* | LP | 12 | Lanping, Yunnan | 99.42 | 26.46 | H4 |
| *P. utilis* | MG | 12 | Maguan, Yunnan | 104.39 | 23.02 | H2 |
| *P. utilis* | ML | 12 | Muli, Sichuan | 101.25 | 27.97 | H10 |
| *P. utilis* | MN | 2 | Mianning, Sichuan | 102.28 | 28.85 | H2 |
| *P. utilis* | NH | 10 | Nanhua, Yunnan | 101.01 | 25.33 | H5 |
| *P. utilis* | QJ | 7 | Qujing, Yunnan | 103.91 | 25.36 | H11 |
| *P. utilis* | QL | 6 | Qianlingshan, Guiyang, Guizhou | 106.69 | 26.60 | H9, H12 |
| *P. utilis* | SZ | 12 | Shizong, Yunnan | 103.98 | 24.82 | H2 |
| *P. utilis* | TM | 15 | Tongmai, Linzhi, Xizang | 94.79 | 30.27 | H3 |
| *P. utilis* | WM | 14 | Wumeng, Luquan, Yunnan | 102.83 | 26.02 | H2 |
| *P. utilis* | WN | 15 | Weining, Guizhou | 104.11 | 26.74 | H13 |
| *P. utilis* | WX | 10 | Weixi, Yunnan | 99.02 | 27.80 | H5, H14 |
| *P. utilis* | XC | 11 | Xichang, Sichuan | 102.35 | 27.70 | H15 |
| *P. utilis* | XCX | 9 | Xichou, Yunnan | 104.79 | 23.38 | H2, H16 |
| *P. utilis* | XJ | 15 | Xinjie, Wenshan, Yunnan | 104.00 | 23.20 | H2, H17 |
| *P. utilis* | XP | 2 | Xinping, Yunnan | 101.95 | 23.96 | H2 |
| *P. utilis* | XS | 4 | Xishan, Kunming, Yunnan | 102.66 | 25.04 | H5, H19 |
| *P. utilis* | YG | 15 | Yigong, Linzhi, Xizang | 94.79 | 30.27 | H3 |
| *P. utilis* | YX | 12 | Yuexi, Sichuan | 102.45 | 28.76 | H15 |
| *P. utilis* | YY | 4 | Yanyuan, Sichuan | 101.46 | 27.60 | H4 |
| *P. utilis* | YS | 15 | Yanshan, Wenshan, Yunnan | 104.42 | 23.69 | H2 |
| *P. utilis* | ZX | 3 | Zhenxiong, Yunnan | 104.86 | 27.45 | H13 |

Appendix 2 The sequences downloaded from GenBank.

| Species | ITS | cpDNA | References |
| --- | --- | --- | --- |
| *Oemleria cerasiformis* | AF318715 | KY419923 | Bortiri *et al.*, 2001; Zhang *et al.*, 2017 |
| *Exochorda racemosa* | AF318740 | KY420012 | Bortiri *et al.*, 2001;  Zhang *et al.*, 2017 |
| *Kerria japonica* | DQ886360 | / | Potter *et al.*, 2007 |
| *K. japonica* var. *pleniflora* | KC156645 | / | Unpublished |
| *Neviusia cliftonii* | MF963992 | / | Thornhill *et al.*, 2017 |
| *Coleogyne ramosissima* | MF963875 | / | Thornhill *et al.*, 2017 |
| *Rhodotypos scandens* | AY177141 | / | Bortiri *et al.*, 2006 |
| *Sorbaria sorbifolia* | JQ041771 | / | Unpublished |
| *Chamaebatiaria millefolium* | DQ886358 | / | Potter *et al.*, 2007 |
| *Adenostoma fasciculatum* | DQ886359 | / | Potter *et al.*, 2007 |

Bortiri, E., Oh, S.H., Jiang, J., Baggett, S., Granger, A., Weeks, C., Buckingham, M., Potter, D. & Parfitt, D.E. (2001). Phylogeny and systematics of *Prunus* (Rosaceae) as determined by sequence analysis of ITS and the chloroplast trnL-trnF spacer DNA. *Systematic Botany*, 26, 797-807.

Bortiri, E., Heuvel, B.V. & Potter, D. (2006). Phylogenetic analysis of morphology in *Prunus* reveals extensive homoplasy. *Plant Systematics & Evolution*, 259, 53-71.

Potter, D., Eriksson, T., Evans, R.C., Oh, S., Smedmark, J.E.E., Morgan, D.R., Kerr, M., Robertson, K.R., Arsenault, M. & Dickinson, T.A. (2007). Phylogeny and classification of Rosaceae. *Plant Systematics & Evolution*, 266, 5-43.

Thornhill, A.H., Baldwin, B.G., Freyman, W.A., Nosratinia, S., Kling, M.M., Morueta-Holme, N., Madsen, T.P., Ackerly, D.D. & Mishler, B.D. (2017). Spatial phylogenetics of the native California flora. *Bmc Biology*, 15, 96.

Zhang, S.D., Jin, J.J., Chen, S.Y., Chase, M.W., Soltis, D.E., Li, H.T., Yang, J.B., Li, D.Z. & Yi, T.S. (2017). Diversification of Rosaceae since the Late Cretaceous based on plastid phylogenomics. *New Phytologist*, 214, 1355-1367.

Appendix 3 Selected bioclimatic variables with low correlations (r < 0.9) used in ecological niche modelling for different *Prinsepia* species.

| Species | *P. sinensis* | *P. uniflora* | *P. utilis* & *P. scandens* |
| --- | --- | --- | --- |
| Variable* | Bio1 | Bio2 | Bio2 |
|  | Bio2 | Bio3 | Bio3 |
|  | Bio3 | Bio4 | Bio4 |
|  | Bio4 | Bio6 | Bio8 |
|  | Bio8 | Bio8 | Bio9 |
|  | Bio12 | Bio12 | Bio12 |
|  | Bio15 | Bio13 | Bio13 |
|  |  | Bio14 | Bio14 |
|  |  | Bio15 | Bio15 |
|  |  | Bio16 |  |

*The Description of the bioclimatic variables can be found at the website (http://worldclim.org/bioclim).
